# Effect of Gallium as an Additive Over Corresponding Ni–Mo/γ-Al_2_O_3_ Catalysts on the Hydrodesulfurization Performance of 4,6-DMDBT

**DOI:** 10.3389/fchem.2022.865375

**Published:** 2022-03-15

**Authors:** Meng Huang, Wenbin Huang, Anqi Li, Han Yang, Yijing Jia, Zhiqing Yu, Zhusong Xu, Xiaohan Wang, Yasong Zhou, Qiang Wei

**Affiliations:** ^1^ State Key Laboratory of Heavy Oil Processing, College of Chemical Engineering and Environment, China University of Petroleum, Beijing, China; ^2^ Fushun Research Institute of Petroleum and Petrochemicals, SINOPEC, Fushun, China

**Keywords:** Ga modification, HDS catalyst, 4,6-DMDBT conversion rate, DDS route selectivity, active phase

## Abstract

Experiments were carried out to research the different contents of Ga_2_O_3_ modification effects on the hydrodesulfurization (HDS) performance of 4,6-dimethyldibenzothiophene (4,6-DMDBT) catalyzed by the stepwise impregnation method. Characterization techniques such as XRD, BET, HRTEM, NH_3_-TPD, and Py-FTIR were performed to determine the effects of each modification of the catalyst by Ga on the properties of the prepared supports and catalysts. The catalytic effect of gallium is reflected in the fact that the empty d-orbitals of Ga elements participate in the formation of molecular orbitals in the active center and change their orbital properties, thus generating a direct desulfurization active phase suitable for complex sulfides for endpoint adsorption. The characterization results indicated that the introduction of Ga_2_O_3_ with appropriate content (2 wt.%) promoted Ni and Mo species to disperse uniformly and doping of more Ni atoms into the MoS_2_ crystals, which also increased the average stacking number and the length of MoS_2_. As a result, more NiMoS active phases were favored to form in the system. The specific surface area and the amounts of acid sites were increased, facilitating the adsorption of reactant molecules and the HDS reactions. The HDS results also suggested the effects of Ga modification play a very important role in the catalytic performance of the corresponding catalysts. The catalyst Ga–Ni–Mo/Al_2_O_3_ exhibited the highest conversion rate towards 4,6-DMDBT HDS when the amount of Ga_2_O_3_ loading was 2 wt.% with an LHSV of 2.5 h^−1^ at 290°C and Ga modification also can effectively improve the direct desulfurization (DDS) route selectivity in varying degrees.

## Introduction

With the increase in global environmental pollution and environmental laws and regulations in various countries becoming stricter, exhaust from the combustion of sulfide in automotive diesel has become one of the important sources of pollution (Wang et al., 2017; Liu et al., 2018; Weng et al., 2020; Zeng et al., 2021). The Environmental Protection Agency (EPA) and E.U. stipulate that the sulfur content should not exceed 10 and 15 ppm (Kulkarni and Afonso, 2010; Liu et al., 2020; Guo et al., 2021). Therefore, low sulfurization of diesel fuel and achieving deep desulfurization of diesel fuel have become a key issue in hydrodesulfurization (Humadi et al., 2021). At present, diesel desulfurization technologies being investigated at home and abroad include adsorptive desulfurization (ADS), oxidative desulfurization (ODS), biodesulfurization (BDS), and hydrodesulfurization (HDS). ODS is a technology for oxidizing heavy sulfides by adding one or two oxygen atoms to sulfur using a suitable oxidant at low temperature and pressure; however, the chosen oxidant is not always selective, and the selective solvent for the extraction of sulfur compounds is not necessarily suitable either (Ali et al., 2006). The basic principle of ADS is to use adsorbents to adsorb sulfur compounds in diesel oil so as to remove sulfides from diesel (Selvavathi et al., 2009). However, adsorption desulfurization is difficult to regenerate, and most adsorbents are not highly selective for sulfides such as 4,6-DMDBT, which are difficult to hydrotreat (Jayaraman et al., 2004). BDS is a new technology for the removal of bound sulfur from sulfur-containing heterocyclic compounds in petroleum using aerobic and anaerobic bacteria at atmospheric pressure and temperature, with promising applications (Mohebali and Ball, 2016). Nevertheless, the desulfurization rates of biocatalysts and the ability of organic sulfides limit their large-scale commercialization (Monticello, 2001). Based on the challenges of the abovementioned three technologies, HDS remains the most widely used technology in the world, which is a heterogeneous and conventional hydrogenation reaction (Chandra Srivastava, 2012). Compared with the former three, it has longer catalyst lifetime and stronger catalyst adaptability to the feed and has the advantage of a higher desulfurization rate for HDS (Huang et al., 2018; Weng et al., 2020). However, HDS cannot effectively remove low-reactive sulfur compounds, such as dibenzothiophene (DBT) and its derivatives, especially 4,6-DMDBT. In order to effectively remove 4,6-DMDBT, two pathways, direct desulfurization (DDS) and hydrodesulfurization (HYD), have been studied by many scholars (Gates and Topsøe, 1997; Li et al., 2019). Studies have shown that unsubstituted 4,6-DMDBT HDS reaction is more dependent on the DDS path, where it not only reacts faster but also results in desulfurization under the premise of ensuring that the aromatic rings do not increase. However, it is limited by its steric hindrance of the substituents (Yin et al., 2013; Wang et al., 2020). In order to design and prepare a highly active hydrodesulfurization catalyst, Okamoto and Kubota (2003) synthesized SiO_2_-, TiO_2_-, ZrO_2_-, and Al_2_O_3_-supported catalysts using the CVD technique and found that the activity of hydrodesulfurization is positively correlated with the amount of CoMoS phases. Wagenhofer et al. (2020) thought that unsupported Ni–Mo sulfides react more rapidly than Al_2_O_3_-supported catalysts on the rate of hydrodesulfurization. Naboulsi et al. (2017) considered that the DDS route is a mainly hydrodesulfurization route than HYD when dual mesoporous titania is used as a support. Due to the high OH concentration on the surface of the support, an inherent Brönsted acid center is formed that is conducive to direct desulfurization through isomerization and disproportionation reactions. In addition, the exploration of support modification has not stopped, and composite supports such as SiO_2_–TiO_2_ (Gallegos–Hernández et al., 2020), SiO_2_–Al_2_O_3_ (Xu et al., 2017), ZrO_2_–Al_2_O_3_ (Baston et al., 2015; Díaz–García et al., 2017), and MgO_2_–Al_2_O_3_ (Rana et al., 2007; Guevara-Lara et al., 2010; Vázquez–Garrido et al., 2019) are still a hot topic for the majority of scholars. However, due to its low cost, easy industrialization, and high surface area, as well as excellent thermal, mechanical, and chemical stability, γ-Al_2_O_3_ is still the most widely used carrier for hydrodesulfurization catalysts (Egorova, 2004; Wang et al., 2016).

It has been proposed that most hydrotreating reactions take place at the MoS_2_ edge and HDS has been found to occur at the corner sites (Abrams et al., 1996; Eijsbouts, 1997; Kasztelan et al., 1984; Kasztelan, 1990). Schuit and Gates (1973) believed that the S-atom in the upper layer is chemically bonded to the Mo-atom in the immediate lower layer. When the Mo atoms are reduced from Mo^5+^ to Mo^3+^, the S atoms move out of the surface, thus forming active sites. The cofactor Ni enters into the surface structure of the alumina carrier and induces the formation of a tetrahedral structure of the secondary Al atoms. Each S atom is bonded to two Mo atoms, and when the S atoms are removed, the S hole formed releases the two Mo atoms and causes one of them to form an adsorption site. The performance of the catalyst is directly related to the number of active sites with high catalytic performance. For the NiMoS active phase, Ni mainly contributes to the associated generation of sulfhydryl (SH) groups and their selective incorporation at the MoS_2_ slab edge (Copéret, 2013; Schüth, 2009). When metal elements with suitable electronic structures are introduced into Ni–Mo/Al_2_O_3_ bimetallic catalysts, the active phase of Ni–Mo–S is affected and the concentration of active centers is increased; thus, the activity of the catalyst for hydrodesulfurization reaction is enhanced (Manoli et al., 2004; Varga et al., 2017). Ferdous et al. (2005) suggested the increased activity is related to the decrease of average slab length and the increase of MoS_2_ edge along with angular atom dispersion. More importantly, it can promote the formation of more type II NiMoS active phases (Zhou et al., 2017; Zhou et al., 2018; Tanimu and Alhooshani, 2019). It is generally believed that there is a great correlation between the numbers of type II NiMoS active phases and the catalytic activity of the catalyst (Chen et al., 2013). Research shows that the metal–support interaction (MSI) is crucial for electron transfer between the metal and support when the catalysts support multiple metals (Ning et al., 2017). To explore the effect of metal additives on the activity of HDS, a large number of polymetallic catalysts supported on Γ-Al_2_O_3_ have been investigated, such as Fe (Liu et al., 2021), Zn, Ru, and Ir (Niquillerothlisberger and Prins, 2006), and even noble metal Pt (Liu et al., 2021) and its alloys Pt–Pd (Niquillerothlisberger and Prins, 2006). Pt is favored by scholars over other metals because it has more advantages in providing active hydrogen species, giving the catalyst superior hydrogenation performance. However, the sulfur resistance of Pt-based catalysts is still a major problem that plagues practical applications and needs to be enhanced. In recent years, the addition of Ga as a metal additive for the synthesis of HDS catalysts has started to attract attention due to the advantage of circumventing the high cost of precious metals (Altamirano et al., 2005). Gallium ions not only have a high affinity for tetrahedral sites of alumina but also change the ratio of tetrahedral to octahedral species of Ni (Co) that can participate in the MoS_2_ decoration (Cimino et al., 1975; Jacono et al., 1977). Moreover, people have found that the modification of gallium on the surface will not only change the morphology of the MoS_2_ slab promoted by active metal Ni but also enhance the vulcanization of Mo species (Zhou et al., 2017). Altamirano et al. (2005) observed the addition of Ga increased the activity of the NiMo catalyst and affected the reaction rate of the HYD route and DDS route in different degrees. Ga has the outer electron arrangement of 4s^2^4p^1^ and has an outer orbit similar to that of Ni (3d^8^4s^2^) and Mo (4d^5^5s^1^). As a result, the empty orbitals participate in the formation of active center molecular orbitals and modify the morphology of Ni–Mo–S active phases (Altamirano-Sanchez et al., 2008; Zepeda and Pawelec, 2012; Zhou et al., 2018). Nevertheless, for HDS activity, there is no clear and reasonable explanation about the additional amount of metal content and reaction conditions.

Considering that in this context, a series of catalysts with the Ga content ranging from 2 to 6 wt.% were prepared by the stepwise impregnation method. A succession of characterizations (XRD, BET, TEM, NH3-TPD, and Py-FTIR) were carried out to investigate the effect of Ga loading on the physical and chemical properties of the Ni–Mo/γ-Al_2_O_3_ catalyst. The hydrodesulfurization reaction was evaluated using 4,6-DMDBT as the probe molecule, and the effects of Ga loading and reaction conditions (temperature and liquid hourly space velocities) on the hydrodesulfurization conversion and DDS selectivity of 4,6-DMDBT were examined.

## Experimental

### Preparation of the Supports

First, γ-Al_2_O_3_ was prepared by the strip extrusion method with the following process conditions: pseudo-boehmite (Shandong Aluminum Corporation) and deionized water were mixed thoroughly at a mass ratio of 1:1, and then, 2 wt.% sesbania powder (Shandong Xunda Chemical Group Co., Ltd.) and 5 wt.% nitric acid (HNO_3_, Aladdin, 65%) were added. The mixture was extruded by using an extruding machine at 30 MPa pressure, and the extruded strips were shaped into a clover type with a diameter of 1.5 mm. The supports was naturally dried in a place of protection from light and ventilation for 24 h and then dried in a drying oven at 120°C for 12 h, and finally, the required γ-Al_2_O_3_ supports were obtained in a muffle furnace at a heating rate of 2°C min^−1^, kept at 500°C for 4.0 h, and naturally cooled to room temperature. The obtained supports were sieved into particles with the size of 20–40 meshes.

### Preparation of the Ni–Mo/γ-Al_2_O_3_ Catalysts

The incipient wetness co-impregnation method was used to synthesize Ni–Mo/γ-Al_2_O_3_ catalysts, and the active metal loading was MoO_3_:16 wt.% and NiO:4 wt.%, respectively. The specific preparation process is as follows: a certain mass of ammonium heptamolybdate tetrahydrate [(NH_4_)_6_Mo_7_O_24_⋅4H_2_O, Aladdin, ≥99.8%] was added to an appropriate amount of deionized water and ammonia to dissolve it completely, and then, nickel nitrate hexahydrate [Ni(NO_3_)_2_⋅6H_2_O, Aladdin, ≥99.8%] solution was added and stirred uniformly to obtain the nickel–molybdenum co-impregnated solution. The prepared Ni–Mo co-impregnation solution was evenly added dropwise to the alumina surface, and the samples were naturally dried at room temperature for 24 h, then dried in an oven for 6 h, and finally, heated to 500°C at a rate of 2°C min^−1^ in a muffle furnace kept at a constant temperature for 4.0 h.

### Preparation of the Ga-Modified Ni–Mo/γ-Al_2_O_3_ Series Catalysts

A series of Ga-modified Ni–Mo/γ-Al_2_O_3_ catalysts were prepared using the stepwise impregnation method, in which the Ga_2_O_3_ loading amount was 2 wt.%, 4 wt.%, and 6 wt.%, respectively, and the specific steps were as follows: gallium nitrate (Ga(NO_3_)_3_⋅H_2_O, Aladdin, ≥99.5%) was dissolved in the deionized water, and then, the solution was loaded using incipient wetness impregnation on the Ni–Mo/Al_2_O_3_. The sample was dried in air for 24 h and then dried in an oven at 120°C for 4 h. Finally, it was heated to 500°C at 2°C min^−1^ in a muffle furnace, with a constant temperature for 4.0 h. The obtained catalysts were denoted as SCal (2 wt.% Ga_2_O_3_), Scam (4 wt.% Ga_2_O_3_), and SCah (6 wt.% Ga_2_O_3_) depending on Ga_2_O_3_ content from low to high.

### Material Characterization

All catalyst samples synthesized were characterized on a PANalytical advanced powder diffractometer using Cu Kα radiation with an accelerating voltage of 40 kV and current of 40 mA in the 2θ interval of 5–80 °*via* a scanning rate of 0.2 °s^−1^, by which the patterns of X-ray diffraction (XRD) were recorded. The surface areas of the tested samples were carefully calculated by using the Brunauer–Emmett–Teller (BET) equation in the relative pressure (P/P0) range of 0.05–0.30. The pore diameter distributions and pore volumes of all the investigated samples were calculated using the Barrett–Joyner–Halenda (BJH) method from the N_2_ adsorption isotherms. The catalysts were sulfided in a JQ-III fixed-bed microreactor with 2 wt.% CS_2_ cyclohexane solution at 320°C for 4 h. Afterward, the investigated catalysts were taken on a Philips Tecnai G2 F20 instrument with an acceleration voltage of 200 keV to obtain the images of active phases *via* a high-resolution transition electron microscope (HRTEM). To analyze the results of the average slab length and average stacking number of MoS_2_, no less than 300 MoS_2_ slabs were counted using the equations reported elsewhere (Ortega–Domínguez et al., 2015; W. Zhou et al., 2017).

$$\mathrm{Average}\;\mathrm{slab}\;\mathrm{length}\;\bar{L}=\sum_{i=1}^{n}n_{i}l_{i}/\sum_{i=1}^{n}n_{i},$$ (1)

$$\;\mathrm{Average}\;\mathrm{stacking}\;\mathrm{number}\;\bar{N}=\sum_{i=1}^{n}n_{i}N_{i}/\sum_{i=1}^{n}n_{i},$$ (2)

where *l*_*i*_ is the length of the slab, *n*_*i*_ is the number of slabs with length *l*_*i*_, and *N*_*i*_ is the number of layers in slab *i*.

The acidity of the catalysts was assessed by NH_3_ temperature programmed desorption (NH_3_-TPD), which was performed on an Auto Chem II 2920 automatic chemical adsorption instrument. All catalysts were pretreated in an Ar and air mixture (v:v = 3:1) at 773k for 60 min and then cooled to 373 k in Ar flow and adsorbed NH_3_ for 40 min. Then, the physically adsorbed NH_3_ was removed in a continuous Ar flow for 90 min. The reactor temperature was then programmed to increase with a heating rate of 10°Cmin^−1^ for 65 min, and the amount of desorbed NH_3_ was detected by using a continuous effluent gas monitor with a thermal conductivity detector (TCD). After the IR spectra were recorded on a Magna 560 FT-IR analyzer, pyridine-adsorbed Fourier transform infrared measurements (Py-FTIR) was used to assess the acidity properties of all the samples by using pyridine as a probe molecule. Firstly, the sample was dehydrated for 3 h at 623 K under 10^−2^ Pa vacuum. Second, to obtain the saturated adsorption of the Py-FTIR spectrum, pure pyridine vapor was added to the measured samples at room temperature for 30 min. Finally, the adsorbed pyridine was evacuated, respectively, at 523k and 623k for an hour so as to obtain the desorbed Py-FTIR spectra.

### Catalyst Assessment

4,6-DMDBT was used as a probe to assess the HDS performances of SCal, Scam, and SCah catalysts. On the fixed-bed reactor whose length was 200 mm and inner diameter was 8 mm, 2.0 g of catalyst was loaded with a particle size of 20–40 meshes. In the first place, the pre-sulfidation of tested catalysts was carried out with 2.2 wt.% CS_2_ cyclohexane solution at 330°C and 4 MPa with H_2_/oil (v/v) of 60 for 4 h. Then, the reactor temperature was allowed to drop to the temperature required for the reaction at the previous condition. Stabilization of the 4,6-DMDBT cyclohexane solution at a mass concentration of 1.0% was carried out for 4 h at 4.0 MPa and 270°C, 280, and 290°C, respectively. The H_2_/oil (v/v) was maintained at 120 when all catalysts were evaluated. The collected products of 4,6-DMDBT was determined by the GC–MS technique on an Agilent 4890D gas chromatograph equipped with a 60 m capillary Rtx-1 column (0.25 mm, RESREK). The column temperature was increased from 50 to 300°C at a rate of 15°C min^−1^, while the N_2_ pressure was maintained at 0.3 MPa and the flow rate was 30 ml min^−1^. The 4,6-DMDBT conversions and DDS route selectivity (3,3′-DMBP selectivity) were counted using the following equations, respectively:

$$4,6-\mathrm{DMDBT}\;\mathrm{conversions}\;Con=\left(1-m_{4,6-DMDBT}\right)\times 100\%,$$ (3)

$$\mathrm{DDS}\;\mathrm{Route}\;\mathrm{selectivity}\;S_{DDS}=m_{3,3\prime -DMBP}/\left(1-m_{4,6-DMDBT}\right)\times 100\%,$$ (4)

where *m*_*4,6-DMDBT*_ is the molar fraction of remaining 4,6-DMDBT and *m*_*3,3′-DMBP*_ is the molar fraction of 3,3′-DMBP tested in the liquid products.

## Results and Discussion

### Effect of Ga Modification on Crystalline Structure

**FIGURE 1:**
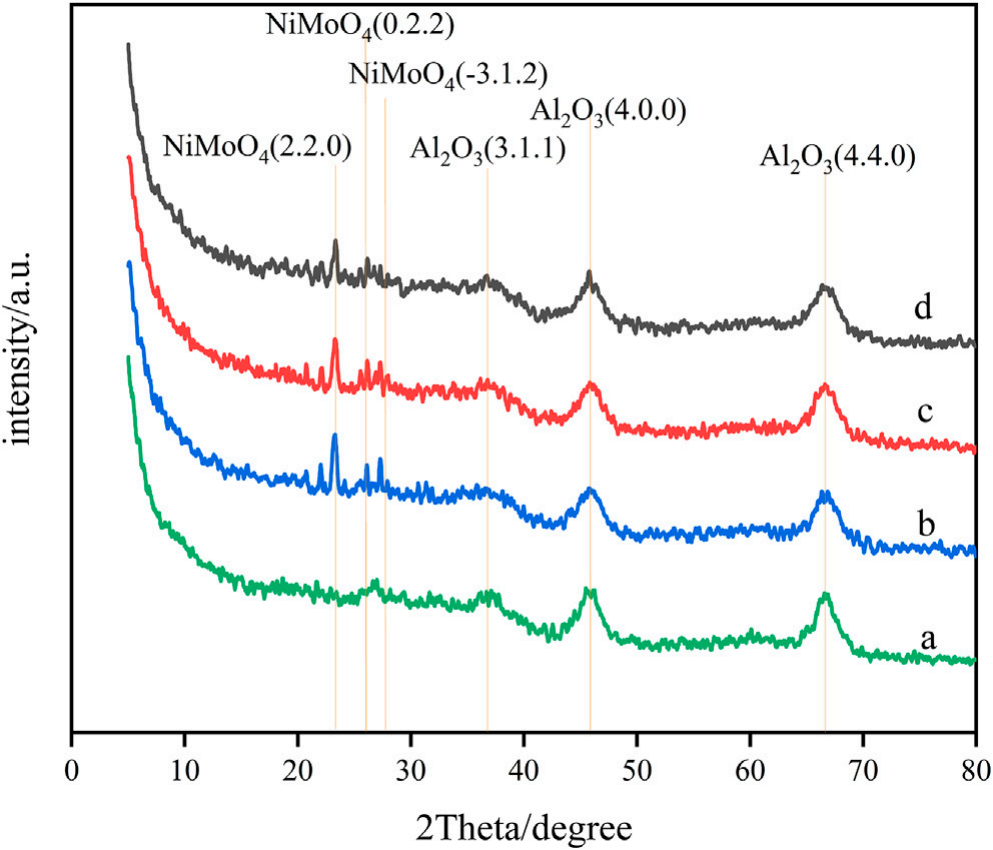
XRD patterns of impregnated Ga-modified Ni–Mo/Al_2_O_3_: (a) Ni–Mo/Al_2_O_3_, (b) SGal, (c) SGam, and (d) SGah.

The spectra of Ni–Mo/Al_2_O_3_ modified by the stepwise impregnation method with different Ga loadings determined by powder X-ray diffraction are shown in Figure 1. It can be seen from the diagrams the characteristic diffraction peaks (4.4.0), (4.0.0), and (3.1.1) of γ-Al_2_O_3_ are almost unchanged, which shows that the crystal structure of γ-Al_2_O_3_ is not affected. No characteristic peaks of Ga_2_O_3_ and other Ga compounds are found in Figure 1, which indicates that Ga_2_O_3_ was highly dispersed on Ni–Mo/Al_2_O_3_ and loaded well. However, diffraction peaks (0.2.2), (-3.1.2), and (2.2.0) of NiMoO_4_ were observed, illustrating that the introduction of Ga_2_O_3_ brought about the agglomeration of Ni and Mo elements. In addition, the characteristic peak intensity of NiMoO_4_ increased with the increase in the content of Ga_2_O_3_, which manifests that the introduction of excessive Ga_2_O_3_ is not conducive to the dispersion of Ni and Mo elements.

### Effect of Ga Modification on Pore Structure

**FIGURE 2:**
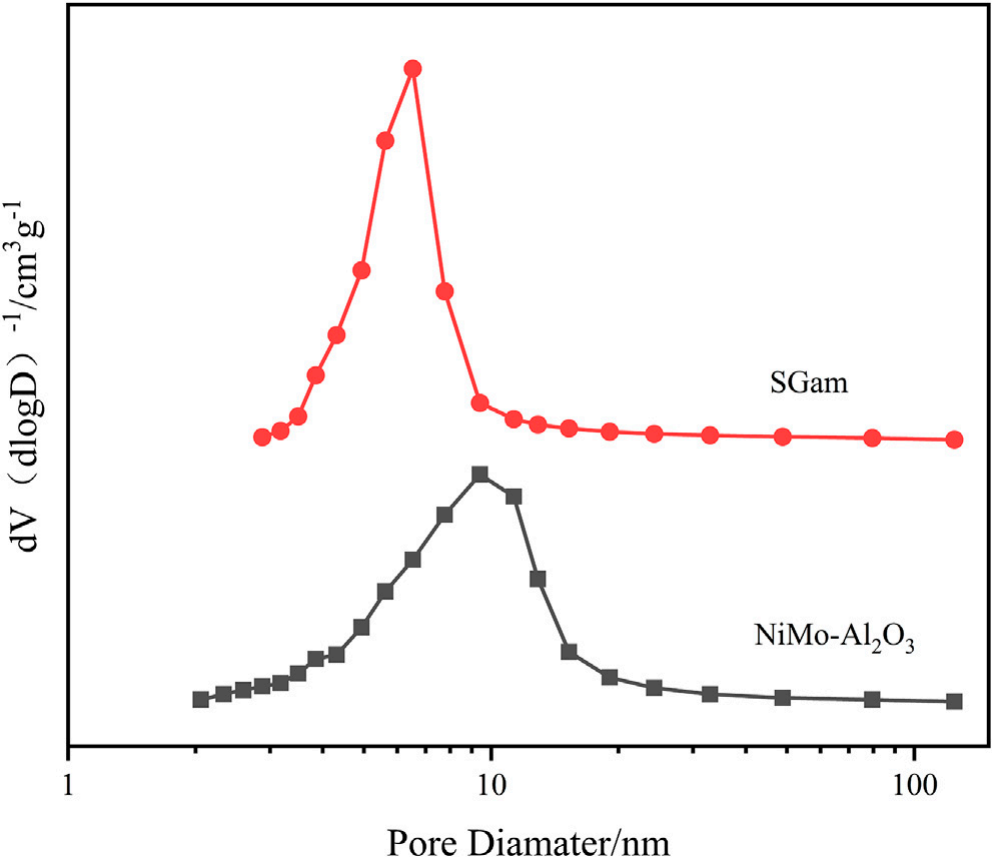
Pore diameter distribution of impregnated Ga-modified Ni–Mo/Al_2_O_3_.

The method of N_2_ physical adsorption–desorption was carried out to confirm the size of the specific surface area, average pore diameter, and pore volume of Ni–Mo/Al_2_O_3_, SGal, SGam, and SGah. The abovementioned textural properties are calculated and displayed about the four synthesized samples in Table 1, and the results of pore size distribution for two samples are recorded in Figure 2. From the data in Table 1, it is clearly found, especially for SGam, that the specific surface area increased from 214 m^2^ g^−1^ to 227 m^2^ g^−1^with the addition of Ga_2_O_3_ in comparison with Ni–Mo/Al_2_O_3_, indicating that the introduced Ga_2_O_3_ had a wide distribution in the inner and outer surface of the catalyst; however, the pore volume and pore size decreased to some extent because parts of Ga_2_O_3_ blocked the pore channel of alumina. It can be seen from Figure 2 that the pore size distribution curve moved to the direction of small pores, and the peak area decreases slightly, which also proved that a large number of pores were blocked or even disappeared with the introduction of gallium.

**TABLE 1:** Pore structural properties of impregnated Ga-modified Ni–Mo/Al_2_O_3_.

Sample	S_BET_, m^2^·g^−1^	V_total_, cm^3^·g^−1^	D, nm
NiMo–Al_2_O_3_	214	0.51	9.5
SGal	218	0.46	6.8
SGam	227	0.45	7.9
SGah	221	0.41	7.5

### Effect of Ga Modification on Morphological Characteristics

**FIGURE 3:**
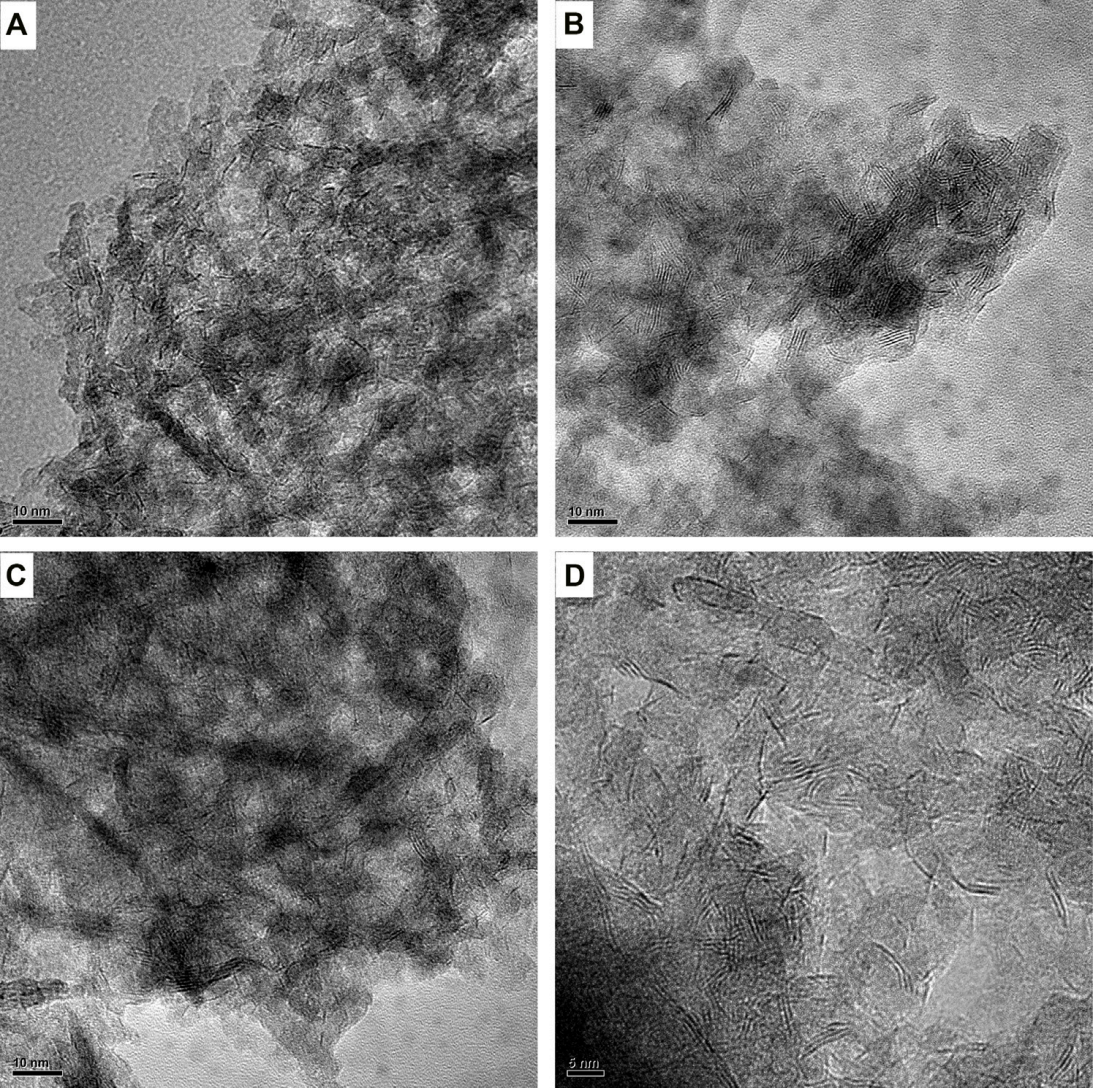
HRTEM of sulfide-impregnated Ga-modified Ni–Mo/Al_2_O_3_: **(A)** Ni–Mo/Al_2_O_3_, **(B)** SGal, **(C)** SGam, and **(D)** SGah.

In order to observe the morphologies, characteristics, and dispersion about the sulfide active phase of Ni–Mo/Al_2_O_3_ and SGax (x stands for l, m, and h) catalysts, the images obtained by HRTEM characterization are shown in Figure 3 **.** At least, 300 MoS_2_ slabs of each catalyst were counted to statistically analyze the results of the average slab length and average stacking number of the active-phase slabs in Table 2. It can be clearly seen from Table 2 that the introduction of Ga contributed to an increase in the average stacking number of MoS_2_ and its change rule kept pace with the increase in Ga loading. Especially, the largest increase of average stacking number for SGal is from 1.1 to 3.6 layers for Ni–Mo/Al_2_O_3_, which displays that the introduction of Ga weakened the Mo–O–Al bond energy and caused a weaker interaction between MoS_2_ and the support, resulting in the formation of more type II Ni–Mo–S reactive phases with high stacking layers. In accordance with the abovementioned regulation, the variation of the average length also has a positive correlation with the Ga_2_O_3_ content. Also, the degrees of average length increase in the following order: SGal (2.4 nm) < Ni–Mo/Al_2_O_3_ (2.7 nm) <SGam (3.1 nm) <SGal (3.3 nm). The average length slightly increased from 2.7 nm for sample Ni–Mo/Al_2_O_3_ to 3.3 nm for sample SGah. This can also be explained by the abovementioned data.

**TABLE 2:** Average length and layer stacks of impregnated Ga-modified Ni–Mo/Al_2_O_3_.

Sample	‾L/nm	‾N
NiMo–Al_2_O_3_	2.7	1.1
SGal	2.4	3.6
SGam	3.1	3.4
SGah	3.3	3.3

### Effect of Ga Modification on Acidity Properties

**FIGURE 4:**
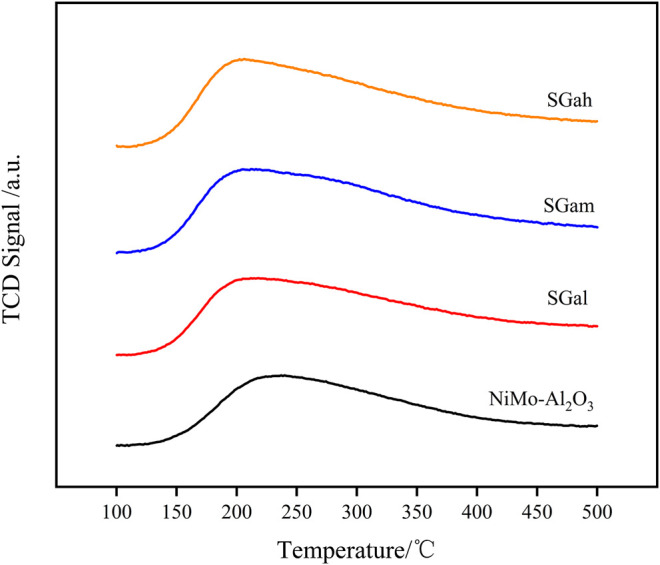
NH_3_-TPD patterns of impregnated Ga-modified Ni–Mo/Al_2_O_3_: (a) Ni–Mo/Al_2_O_3_, (b) SGal, (c) SGam, (d) SGah.

Acidity plays a crucial role in the formation of the active phase and the HDS reaction, so NH_3_-TPD characterization of the series samples was carried out and is displayed in Figure 4. The peak temperature and peak area of the NH_3_ desorption were calculated for each of the investigated samples and are summarized in Table 3 **.** The NH_3_-TPD profiles in Figure 4 reveal the peak temperature of the Ga–Ni–Mo/Al_2_O_3_ catalyst existed mainly between 150 and 300°C, indicating that the catalyst was mainly the prince of weak acid sites and medium–strong acid sites, which was weaker than that of the Ni–Mo/Al_2_O_3_ catalyst. It can be seen from Table 3 that the introduction of Ga made the NH_3_ desorption peak shift to a lower temperature, manifesting that the weak acid sites of the catalyst mainly increased. According to the comparison of peak area, the number of acid sites of the Ni–Mo/Al_2_O_3_ catalyst was increased by introducing Ga_2_O_3_, but it was negatively correlated with introduced Ga_2_O_3_ content. Thus, the acid sites varied significantly for SGal. That phenomenon explained that the acid sites were covered by Ga_2_O_3_, leading to reduction in the degree of increase on the number of acid sites.

**TABLE 3:** NH_3_-TPD results of impregnated Ga-modified Ni–Mo/Al_2_O_3._

Sample	Peak temperature/°C	Peak area
Ni–Mo/Al_2_O_3_	237	1.085
SGal	210	1.423
SGam	207	1.386
SGah	205	1.275

### Effect of Ga Modification on Acid Types and Strength

It is well known that acid types and strength are the key points to decide the performance of the corresponding catalysts. As a consequence, pyridine desorption FTIR analyses were performed at different temperatures, and the results are listed in Table 4, where the specific data of weak and strong Brönsted acid sites (BAS) or Lewis acid sites (LAS) were derived from the information of pyridine desorption at 200 and 350°C, respectively. It can be clearly observed from Table 4 that the introduction of 2 wt.% Ga_2_O_3_ enhanced the weak B-acid, and the B-acid enhancement facilitates the desulfurization of 4,6-DMDBT through the isomeric desulfurization pathway (ISOM). Moreover, after the isomerization of the methyl group on 4,6-DMDBT, the steric hindrance to sulfur atoms decreased significantly, which provided access to direct desulphurization just by hydrogenolysis of sulfur atoms, and the path to remove sulfur was considered to be very ideal. In addition, the introduction of different contents of Ga increased the Lewis acid, among which the weak Lewis acid increased more and the strong Brönsted acid decreased slightly. This is consistent with the characterization of NH_3_-TPD. Except for the enhancement of weak Brönsted acid by SGam, the amount of Ga_2_O_3_ introduced had little effect on the acid type and amount of Brönsted acid and Lewis acid of Ni–Mo/Al_2_O_3_, but the amount of Ga_2_O_3_ introduced increased the total acid amount.

**TABLE 4:** Acidity properties of impregnated Ga-modified Ni–Mo/Al_2_O_3._

Sample	Weak acid sites/μmol·g^−1^				Strong acid sites/μmol·g^-1^			
	B	L	B/L	B + L	B	L	B/L	B + L
Ni–Mo/Al_2_O_3_ SGal	54	106	50.94	160	10	85	11.76	95
	116	217	53.46	333	0	159	0	159
SGam	35	181	19.34	216	0	137	0	137
SGah	35	224	15.63	259	0	165	0	165

### Assessment of Catalytic Activities

**FIGURE 5:**
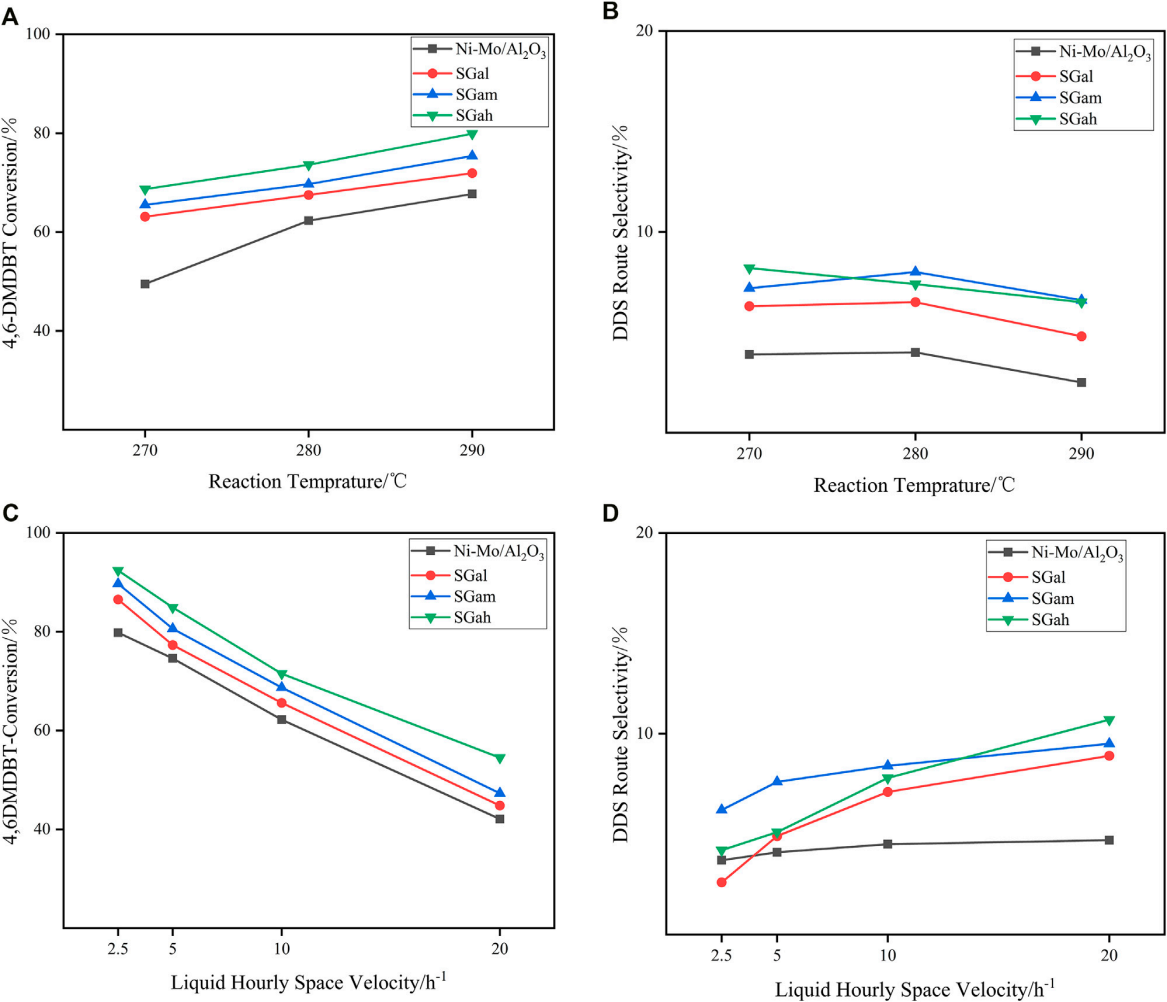
Catalytic performance of impregnated Ga-modified Ni–Mo/Al_2_O_3_: **(A)** 4,6-DMDBT conversion on different reaction temperatures, **(B)** DDS selectivity of 4,6-DMDBT on different reaction temperatures, **(C)** 4,6-DMDBT conversion on different LHSVs at 280°C, and **(D)** DDS selectivity of 4,6-DMDBT conversion on different LHSVs at 280°C.

As is concretely confirmed, though the appropriate content of Ga_2_O_3_ modification can effectively improve the physicochemical properties of the catalysts from the characterization results mentioned above, the favorable conditions for conversion and selectivity at different temperatures need to be explored. Thus, 4,6-DMDBT served as the probe molecules in the assessment of the HDS performance of Ni–Mo/Al_2_O_3_ and SGax series catalysts on a fixed-bed reactor under a total pressure of 4 MPa and the liquid hourly space velocities (LHSVs) of 2.5 h^−1^ at different reaction temperatures (in the range of 270–290°C). The variation of conversions on 4,6-DMDBT at different temperatures is clearly displayed in Figure 5A, suggesting that the activities of all the investigated catalysts increased with the increase in the reaction temperature. These results also pointed out that there was no significant disparity in the conversion of SGax series catalysts with different Ga_2_O_3_ loadings, and the highest conversion rate was obtained when the Ga_2_O_3_ loading was 2 wt.%. In addition, with the increase in reaction temperature, the difference of conversion rate between SGax series catalysts and the Ni–Mo/Al_2_O_3_ catalyst became smaller, which resulted from the increase in the conversion rate of 4,6-DMDBT at high temperature close to the reaction end point.

**SCHEME 1:**
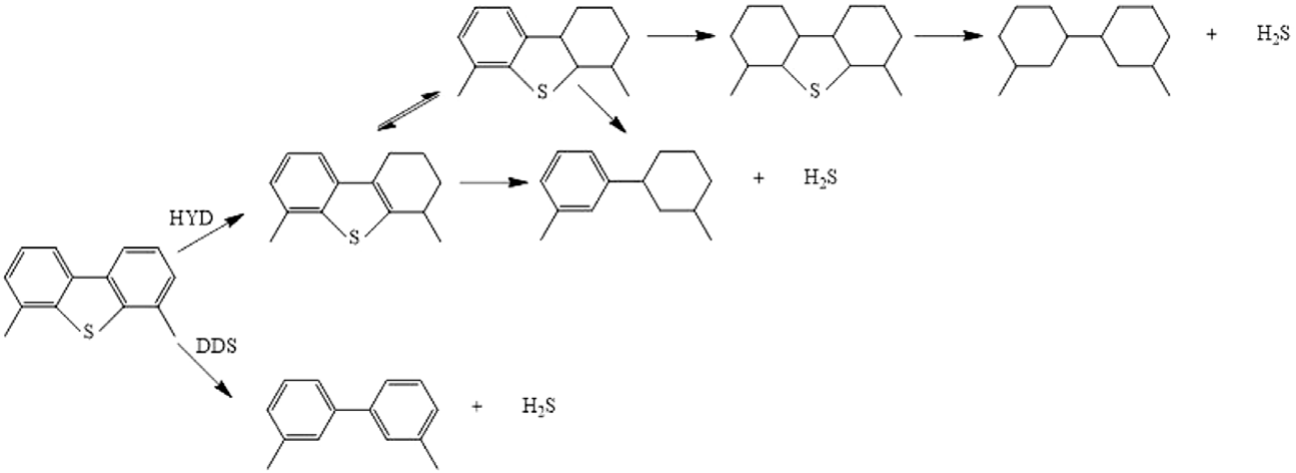
Reaction scheme of HDS for 4,6-DMDBT over Ga–Ni–Mo/Al_2_O_3_ catalysts. HYD, hydrogenation. DDS, direct desulfurization.

Scheme 1 shows the HDY and DDS reaction route over Ga–Ni–Mo/Al_2_O_3_ catalysts separately. HYD pathway desulfurization is a tedious step to form the products 3,3′-dimethylcyclohexanebenzene (3,3′-DMCHB) or 3,3′-dimethylbicyclohexyl (3,3′-DMBCH), while DDS pathway desulfurization is achieved with fewer steps and without excess aromatic rings and produces 3,3′-dimethylbiphenyl (3,3′-DMBP). The DDS route selectivity over different catalysts at the same conditions mentioned above was obtained, and the results are shown in Figure 5B. It can be observed from Figure 5B that, with the increase in reaction temperature, the DDS selectivity of the tested catalysts on the DMDBT decreased, owing to that Ni gradually began to be reduced as the reaction temperature exceeded 290°C. Especially, the metal Ni has a strong hydrogenation performance. The reason mentioned above led to the enhancement of the HYD path. In addition, it was also found that if the reaction temperature is higher than 290°C, the high hydrogenation performance of Ni can even convert a small amount of DDS products into HYD products, but it was generally considered that this reaction will not occur, which was the unique characteristic of Ni-containing hydrotreating catalysts. Further increase in temperature would lead to a profound increase in the proportion of hydrogenation conversion of DDS products to HYD products. In order to properly deal with this situation, the metal promoter can be replaced with the load of Co with low hydrogenation performance, and the amount of the Ni loading or the Ni/Mo atomic ratio ought to be reduced.

The influence of different LHSVs in the investigated series catalysts on the 4,6-DMDBT HDS reaction is shown in Figure 5C. As can be clearly noticed from the figure, with the decrease in LHSVs which meant increasing the residence time of raw materials, the conversion rate of 4,6-DMDBT increased gradually and over the tested catalysts was in the order of Ni–Mo/Al_2_O_3_<SGah < SGam < SGah. When the LHSV decreased gradually, the increasing rate of conversion rates slowed down gradually as the reaction is close to complete conversion. Thus, the conversion rate gap between different Ga_2_O_3_ loading catalysts was bridged.

Figure 5D reveals the transformation about selectivity of DDS route by stepwise impregnation of the Ga–Ni–Mo/Al_2_O_3_ catalyst with different LHSVs on 4,6-DMDBT at a reaction temperature of 280°C. As it is vividly shown in Figure 5D, the gallium-modified catalysts exhibited higher DDS route selectivity than the Ni–Mo/Al_2_O_3_ catalyst at high LHSV. However, the DDS route selectivity of SGax series catalysts for 4,6-DMDBT exhibited a negative correlation with the variation of LHSV. With the decrease in LHSV, the DDS selectivity of SGax series catalysts for 4,6-DMDBT even decreased to about the corresponding selectivity of the Ni–Mo/Al_2_O_3_ catalyst. On the one hand, the incorporation of Ga improved the average stacking number of the active-phase slabs and generated more type II NiMoS active phases which weekly interact with the support or do not interact with the support at all, and it is considered to be very active (Nikulshin et al., 2014; van Haandel et al., 2015), so that the HYD route got strengthened. On the other hand, the residence time of raw materials became longer while the LHSV decreased, which is beneficial to the HYD route with longer reaction path and slower reaction rate. In addition, because of the strong hydrogenation ability of the reduced metal Ni, a small part of DDS products was also transformed into HYD products. All mentioned above were identified as the reason for which the variation of LHSV had impact on the DDS route selectivity.

## Conclusion

The experiments showed that the introduction of Ga_2_O_3_ with appropriate content (2 wt.%) promoted not only Ni and Mo species to disperse uniformly but also doping of more Ni atoms into the MoS_2_ crystals. In addition, the average stacking number and the length of Mo_2_S were increased. Those mentioned above resulted in the formation of more NiMoS active phases. The specific surface area and the amount of acid sites were increased, facilitating the adsorption of reactant molecules and the hydrodesulfurization reactions. Last but not the least, the catalyst Ga–Ni–Mo/Al_2_O_3_ exhibited the highest conversion rate towards 4,6-DMDBT HDS when the amount of Ga_2_O_3_ loading was 2 wt.% with an LHSV of 2.5 h^−1^ at 290°C, and Ga modification also can effectively improve the DDS route selectivity in varying degrees.

## Data Availability

The original contributions presented in the study are included in the article/Supplementary Material, further inquiries can be directed to the corresponding author.
