# Supplementary figures and images for: Effect of Gallium as an Additive Over Corresponding Ni–Mo/γ-Al2O3 Catalysts on the Hydrodesulfurization Performance of 4,6-DMDBT
Source: Front Chem. 2022 Mar 15;10:865375. doi: 10.3389/fchem.2022.865375 (PMC8965378; doi:10.3389/fchem.2022.865375)

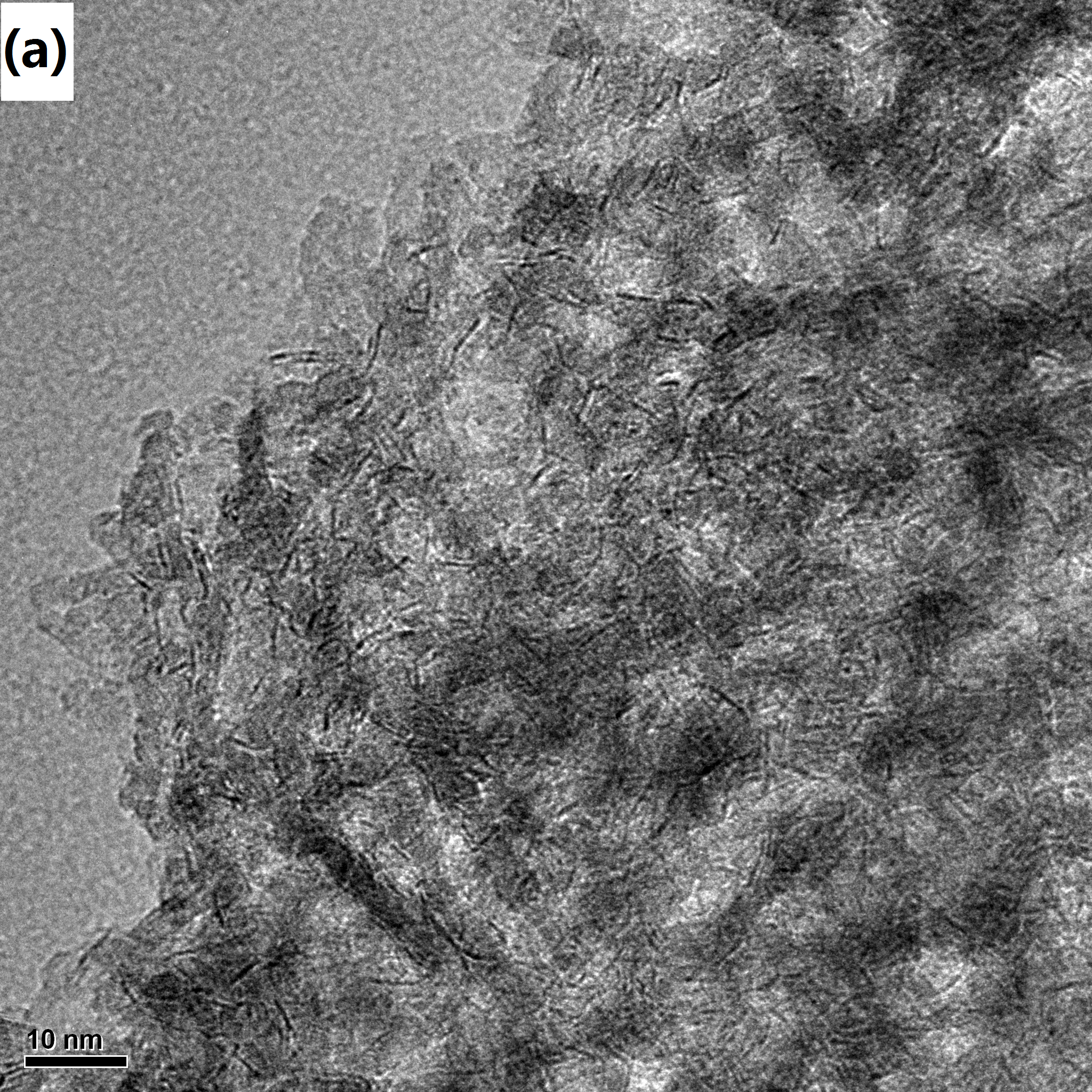

Supplement: Supplementary file 1 [file DataSheet1.ZIP › supplementary material presentation1/FIGURE 3/HRTEM-A.tif]

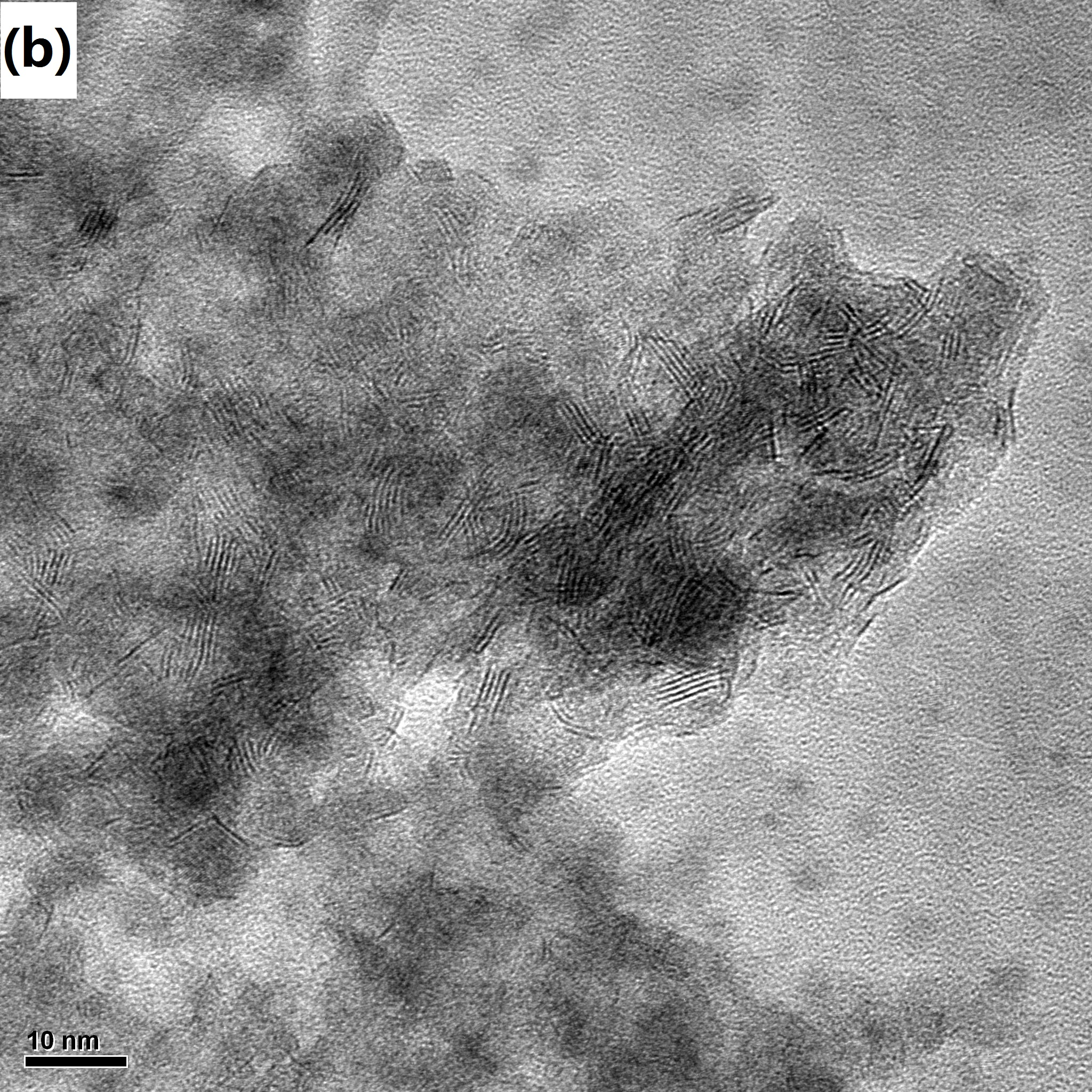

Supplement: Supplementary file 1 [file DataSheet1.ZIP › supplementary material presentation1/FIGURE 3/HRTEM-B.tif]

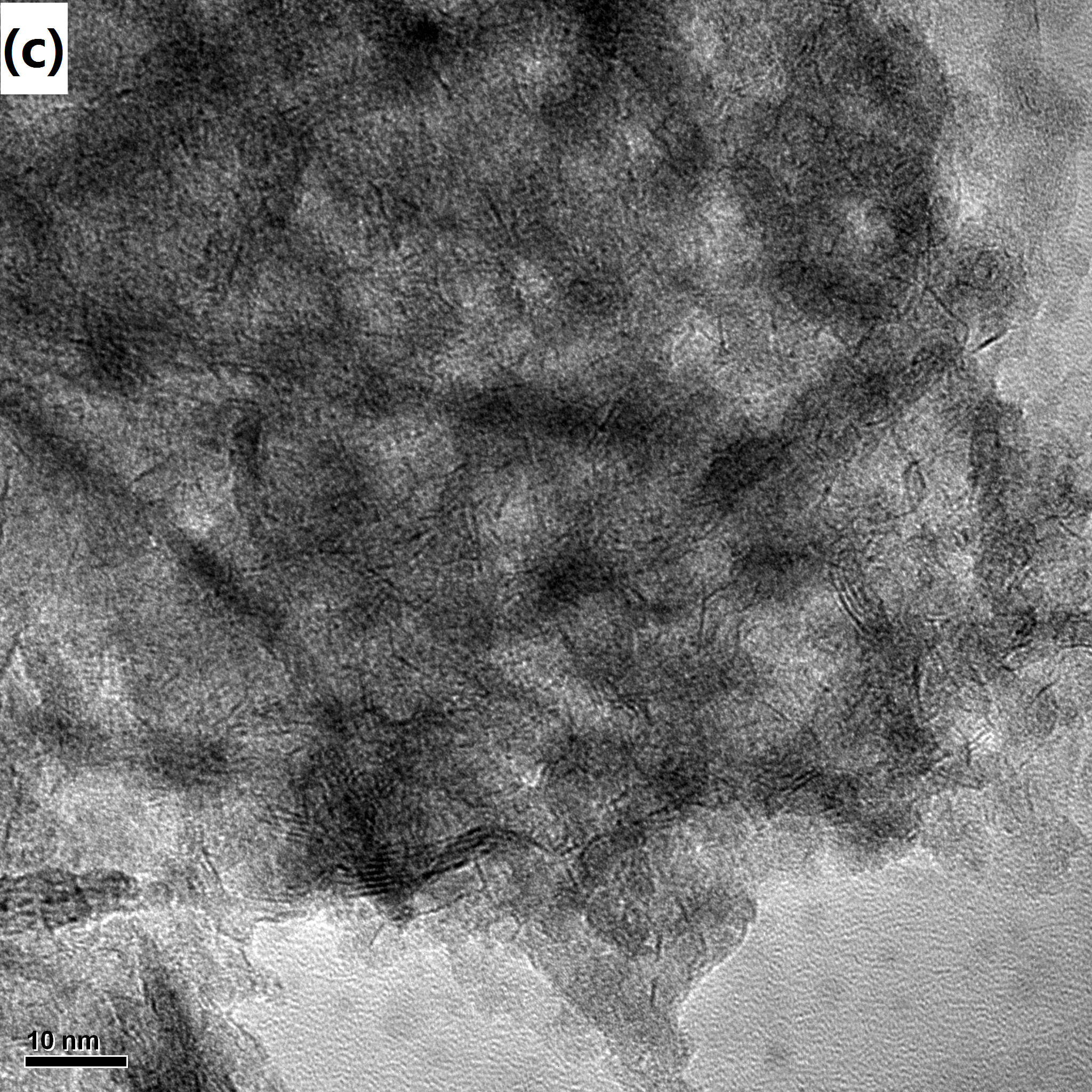

Supplement: Supplementary file 1 [file DataSheet1.ZIP › supplementary material presentation1/FIGURE 3/HRTEM-C.tif]

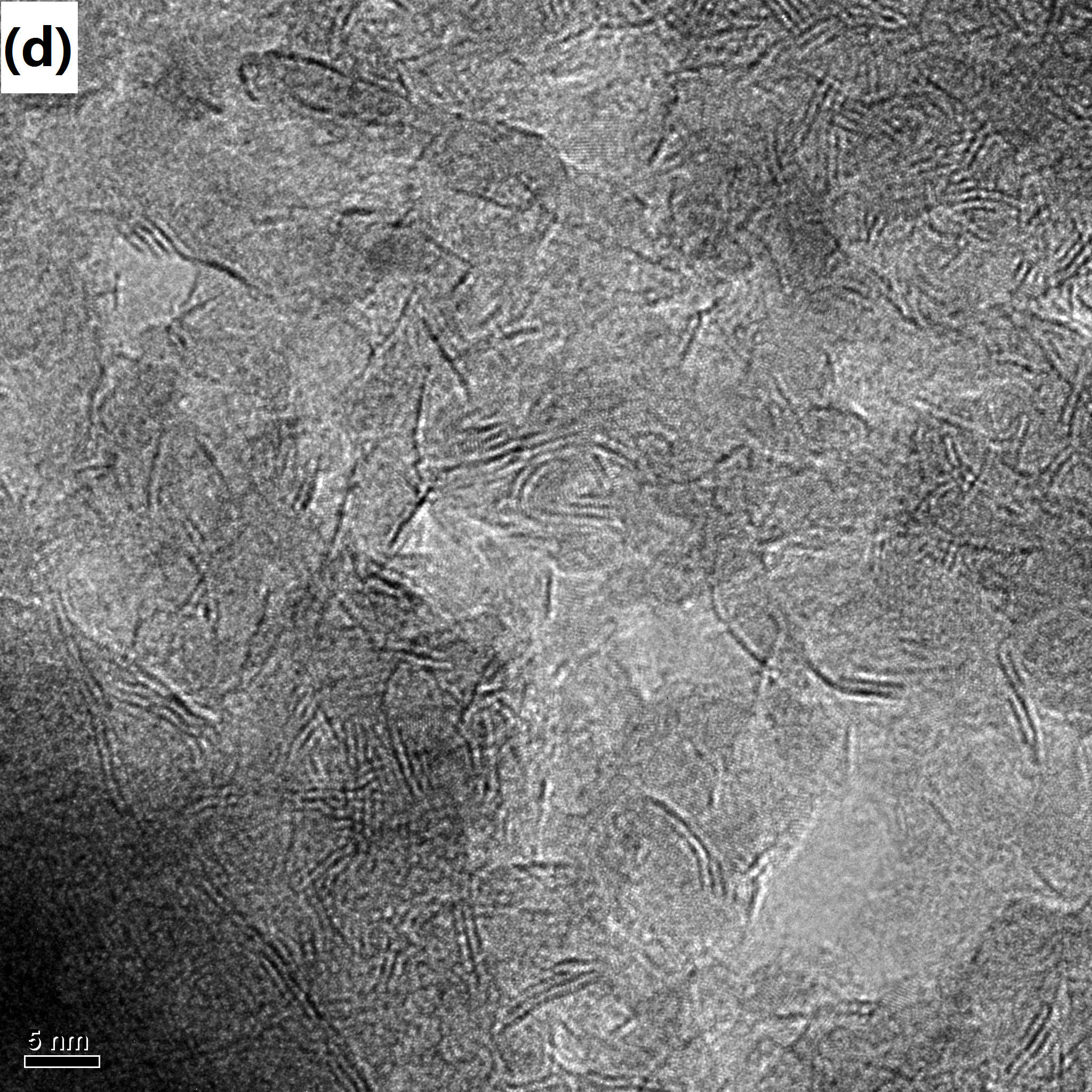

Supplement: Supplementary file 1 [file DataSheet1.ZIP › supplementary material presentation1/FIGURE 3/HRTEM-D.tif]
